# Impact of L-carnitine on plasma lipoprotein(a) concentrations: A systematic review and meta-analysis of randomized controlled trials

**DOI:** 10.1038/srep19188

**Published:** 2016-01-12

**Authors:** Maria-Corina Serban, Amirhossein Sahebkar, Dimitri P. Mikhailidis, Peter P. Toth, Steven R. Jones, Paul Muntner, Michael J. Blaha, Florina Andrica, Seth S. Martin, Claudia Borza, Gregory Y. H. Lip, Kausik K. Ray, Jacek Rysz, Stanley L. Hazen, Maciej Banach

**Affiliations:** 1 Department of Epidemiology, University of Alabama at Birmingham, Birmingham, AL, USA; 2 Department of Functional Sciences, Discipline of Pathophysiology, “Victor Babes” University of Medicine and Pharmacy, Timisoara, Romania; 3 Biotechnology Research Center, Mashhad University of Medical Sciences, Mashhad, Iran; 4 Metabolic Research Centre, Royal Perth Hospital, School of Medicine and Pharmacology, University of Western Australia, Perth, Australia; 5 Department of Clinical Biochemistry, Royal Free Campus, University College London Medical School, University College London (UCL), London, UK; 6 Preventive Cardiology, CGH Medical Center, Sterling, Illinois, USA; 7 The Johns Hopkins Ciccarone Center for the Prevention of Heart Disease, Baltimore, MD, USA; 8 Faculty of Pharmacy, Discipline of Pharmaceutical Chemistry “Victor Babes” University of Medicine and Pharmacy, Timisoara, Romania; 9 University of Birmingham Centre for Cardiovascular Sciences, City Hospital, Birmingham, UK; 10 Department of Primary Care and Public Health, School of Public Health, Imperial College London, UK; 11 Department of Hypertension, Chair of Nephrology and Hypertension, Medical University of Lodz, Poland; 12 Department for Cellular and Molecular Medicine, Lerner Research Institute, Cleveland Clinic, Cleveland, OH, USA

## Abstract

We aimed to assess the impact of L-carnitine on plasma Lp(a) concentrations through systematic review and meta-analysis of available RCTs. The literature search included selected databases up to 31^st^ January 2015. Meta-analysis was performed using fixed-effects or random-effect model according to *I*^*2*^ statistic. Effect sizes were expressed as weighted mean difference (WMD) and 95% confidence interval (CI). The meta-analysis showed a significant reduction of Lp(a) levels following L-carnitine supplementation (WMD: −8.82 mg/dL, 95% CI: −10.09, −7.55, *p* < 0.001). When the studies were categorized according to the route of administration, a significant reduction in plasma Lp(a) concentration was observed with oral (WMD: −9.00 mg/dL, 95% CI: −10.29, −7.72, *p* < 0.001) but not intravenous L-carnitine (WMD: −2.91 mg/dL, 95% CI: −10.22, 4.41, *p* = 0.436). The results of the meta-regression analysis showed that the pooled estimate is independent of L-carnitine dose (slope: −0.30; 95% CI: −4.19, 3.59; *p* = 0.878) and duration of therapy (slope: 0.18; 95% CI: −0.22, 0.59; *p* = 0.374). In conclusion, the meta-analysis suggests a significant Lp(a) lowering by oral L-carnitine supplementation. Taking into account the limited number of available Lp(a)-targeted drugs, L-carnitine might be an effective alternative to effectively reduce Lp(a). Prospective outcome trials will be required to fully elucidate the clinical value and safety of oral L-carnitine supplementation.

In 1963, Kåre Berg discovered lipoprotein(a) [Lp(a)] in human plasma, a low-density lipoprotein (LDL)-like lipoprotein particle with atherogenic and thrombotic properties^1^. Lp(a) is composed by a central LDL-like lipoprotein core particle with apolipoprotein B (apoB) covalently bound to glycoprotein apo(a), resulting in differing structure, physical and chemical properties compared with LDL^1^,^2^. The levels of Lp(a) in human plasma are genetically modulated, and Precocious Coronary Artery Disease (PROCARDIS) study showed that two Lp(a) single nucleotide polymorphisms (SNPs) accounted for 36% of variation in Lp(a) levels^3^. The levels of Lp(a) vary among populations; African descendants have increased Lp(a) levels as compared to Caucasians and Asian descendants^4^. However, different conditions such as diabetes, endocrine disorders, renal and liver failure or the acute-phase response might influence the synthesis or catabolism of Lp(a)^5^,^6^. The development of immunoassays to precisely measure Lp(a) levels in plasma was impeded by the structural complexity and size heterogeneity of Lp(a)^3^.

Many studies have shown that increased plasma Lp(a) levels are associated with coronary artery disease^7^,^8^, peripheral arterial disease^9^, cerebrovascular disease^10^, abdominal aortic aneurysm, aortic valve stenosis and calcification^11^, and venous thromboembolism^12^. Moreover, elevated Lp(a) levels have been found to be associated with increased risks for major adverse cardiac events (MACE)^13^. The atherogenic impact influenced by the effective binding of oxidized phospholipids by Lp(a), the expression of adhesion molecules by Lp(a)^14^, the deposit of Lp(a) in the arterial wall and the prothrombotic effect caused by the homology of apo(a) to plasminogen are potential mechanisms by which Lp(a) raises cardiovascular (CV) risk^15^,^16^. After migration from plasma into the arterial intima, Lp(a) joins to the extracellular matrix through both apo(a) and apoB components, resulting in the growing of the atherosclerotic plaque^17^.

In 2010, the European Atherosclerosis Society (EAS) Consensus Panel recommended a target level for Lp(a) < 50 mg/dL, as a feature of global CV risk^18^. The treatment of choice for the patients with increased Lp(a) levels is niacin or nicotinic acid (1–3 g/day), as recommended by the European Society of Atherosclerosis (EAS) and American Heart Association (AHA)/American Stroke Association (ASA) guidelines^18^,^19^. However, after negative trials with niacin, statins are worldwide recommended to reduce the global CV risk^20^. Moreover, observational studies suggested that the enhanced CV risk associated with increased Lp(a) levels is no longer observed when LDL-C levels are treated to aggressive levels, suggesting intensification of statin therapy and LDL-C lowering as a potential option in subjects with Lp(a) elevation^13^,^20^. Niacin therapy produces side-effects *via* stimulation of prostaglandin D(2) and E(2) synthesis, posing another obstacle to its use for Lp(a) elevation in clinical practice^21^. Therefore, a new therapy to target high Lp(a) levels is considered an important and unmet clinical need^22^. Besides niacin and intensive statin therapy, tibolone has also been investigated in patients with elevated Lp(a) levels^23^. Other drugs currently under development include cholesteryl ester transfer protein (CETP) inhibitors^24^, proprotein convertase subtilisin/kexin type-9 (PCSK9) inhibitory monoclonal antibodies^24^,^25^, mipomersen, an antisense oligonucleotide (ASO) against apoB mRNA^26^, eprotirome, a thyroid analogue^27^, lomitapide, a microsomal triglyceride transfer protein inhibitor^28^, and ASO therapy (ISIS APO(a) Rx) directly against apo(a)^29^,^30^. In addition, there has been a surge of interest to screen natural products (nutraceuticals/functional foods) for their effects on Lp(a) concentration^31^,^32^.

Different studies have indicated that L-carnitine, an amino acid involved in mitochondrial fatty acid oxidation and ATP production, might be associated with reduction in Lp(a) levels^33^. The metabolism of L-carnitine is complex, and involves formation of many short and long chain acylated forms, as well as metabolism to its core structure^31^,^32^,^33^. In addition, there are substantial alterations in the metabolism of L-carnitine depending upon whether it is provided as intravenous supplement, such as during hemodialysis, or as oral supplement^34^. With the latter route of supplementation, gut microbes have been shown to significantly impact L-carnitine metabolism, and to convert it into alternative metabolites, linked to increased atherosclerosis risk^35^,^36^.

Therefore, we aimed to assess the impact of L-carnitine supplementation and type of administration on plasma Lp(a) concentrations through systematic review of literature and meta-analysis of available randomized controlled trials (RCTs).

## Methods

### Search Strategy

This study was designed according to the guidelines of the 2009 preferred reporting items for systematic reviews and meta-analysis (PRISMA) statement^37^. SCOPUS (http://www.scopus.com) and Medline (http://www.ncbi.nlm.nih.gov/pubmed) databases were searched and the search was limited to the randomized controlled studies (RCTs) carried out up to January 31, 2015 investigating the potential effects of L-carnitine supplementation on Lp(a) concentrations. The databases were searched using the following search terms in titles and abstracts (also in combination with MESH terms): (L-carnitine) AND Lp(a), or (L-carnitine) AND Lipoprotein(a). The wild-card term “*” was used to increase the sensitivity of the search strategy. No language restriction was used in the literature search. The search was limited to studies in human. Selected articles were searched to identify further relevant studies. Two reviewers (CS and AS) evaluated each article separately. Disagreements were resolved by agreement and discussion with a third party (MB).

### Study Selection

Original studies were included if they met the following inclusion criteria: (i) being a randomized controlled trial with either parallel or cross-over design, (ii) investigating the impact of L-carnitine on plasma/serum concentrations of Lp(a), and, (iii) presentation of sufficient information on Lp(a) concentrations at baseline and at the end of follow-up in each group or providing the net change values.

Exclusion criteria were: (i) non-randomized trials, (ii) lack of an appropriate control group in the study design, (iii) observational studies with case-control, cross-sectional or cohort design, (iv) lack of sufficient information on baseline or follow-up Lp(a) concentrations, (v) inability to obtain adequate details of study methodology or results from the article or the investigators, and, (vi) the study was ongoing.

### Data extraction

Eligible studies were reviewed and the following data were abstracted: (1) first author’s name, (2) year of publication, (3) study location; (4) study design; (5) number of participants in the L-carnitine and control (in case of randomized design) groups, (6) age, gender and body mass index (BMI) of study participants, (7) baseline levels of total cholesterol, LDL-C, high-density lipoprotein cholesterol (HDL-C), triglycerides, high-sensitivity C-reactive protein (hsCRP) and glucose, (8) systolic and diastolic blood pressures, and, (9) data regarding baseline and follow-up concentrations of Lp(a).

### Quality assessment

A systematic assessment of bias in the included studies was performed using the Cochrane criteria^38^. The items used for the assessment of each study were as follows: adequacy of sequence generation, allocation concealment, blinding, and handling of dropouts (incomplete outcome data), selective outcome reporting, and other potential sources of bias. According to the recommendations of the Cochrane Handbook, a judgment of “yes” indicated low risk of bias, while “no” indicated high risk of bias. Labeling an item as “unclear” indicated an unclear or unknown risk of bias.

### Quantitative Data Synthesis

Meta-analysis was conducted using Comprehensive Meta-Analysis (CMA) V2 software (Biostat, NJ)^39^. Net changes in measurements (change scores) were calculated as follows: measure at end of follow-up − measure at baseline. For single-arm cross-over trials, net change in plasma concentrations of L-carnitine was calculated by subtracting the value after control intervention from that reported after treatment. Standard deviations (SDs) of the mean difference were calculated using the following formula: SD = square root [(SD_pre-treatment_)^2^ + (SD_post-treatment_)^2^ − (2 R × SD_pre-treatment_ × SD_post-treatment_)], assuming a correlation coefficient (R) = 0.5. If the outcome measures were reported in median and inter-quartile range, mean and standard SD values were estimated using the method described by Hozo *et al.*^40^. Where standard error of the mean (SEM) was only reported, standard deviation (SD) was estimated using the following formula: SD = SEM × square root (*n*), where *n* is the number of subjects.

Meta-analysis was performed using either a fixed-effects or random-effect model according to the *I*^*2*^ statistic. *I*^*2*^ values < 50% and ≥ 50% corresponded with the use of fixed-effects and random-effects model, respectively. The generic inverse variance method was used to weight each individual study included in the meta-analysis. Heterogeneity was quantitatively assessed using the *I*^*2*^ index. Effect sizes were expressed as weighted mean difference (WMD) and 95% confidence interval (CI). In order to evaluate the influence of each study on the overall effect size, a sensitivity analysis was conducted using the leave-one-out method (i.e., removing one study each time and repeating the analysis).

### Publication bias

Potential publication bias was explored using visual inspection of Begg’s funnel plot asymmetry, and Begg’s rank correlation and Egger’s weighted regression tests. Duval & Tweedie’s “trim and fill” method was used to adjust the analysis for the effects of publication bias^41^.

## Results

### Search results and trial flow

**Figure 1:**
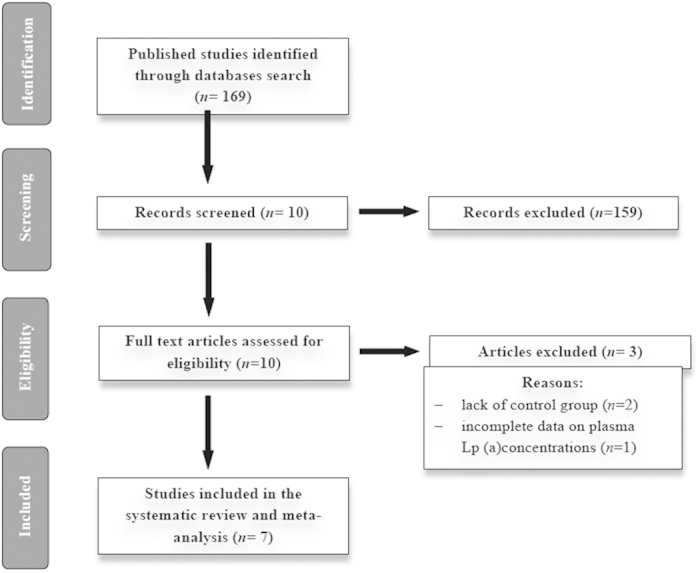
Flow chart of the number of studies identified and included into the meta-analysis.

The initial screening for possible interpretation eliminated the articles whose titles and/or abstracts were clearly insignificant. After evaluation, 7 studies with 8 treatment arms (intravenous administration: 2 eligible studies with 3 treatment arms, oral administration: 5 eligible studies with 5 treatment arms) met the inclusion criteria and were chosen for the final meta-analysis. A study flow chart is presented on Fig. 1.

### Characteristics of included studies

In total, 375 participants were randomized, of whom 200 were assigned to the L-carnitine supplementation group and 175 to the control group. The number of participants in these trials ranged from 15 to 46. Studies were published between 2000 and 2014, and were conducted in the Italy, China and Iran. A range of doses from 1 g 3 times per week to 4 g/day of L-carnitine was administered in the included trials. Duration of L-carnitine supplementation ranged from 1 week to 24 weeks. L-carnitine appeared safe and well-tolerated in all RCTs included in this analysis, with no report of any serious adverse events. Demographic and baseline parameters of the included studies are shown in Table 1.

### Risk of bias assessment

According to the Cochrane Collaboration, a specific tool for assessing risk of bias in each included study comprises judgment of specific features of the study^42^. This involves evaluating the risk of bias as ‘low risk’, ‘high risk or ‘unclear risk’. The final category implies either lack of information or doubt over the potential for bias. There are seven analyzed domains comprising: sequence generation (selection bias), allocation sequence concealment (selection bias), blinding of participants and personnel (performance bias), blinding of outcome assessment (detection bias), incomplete outcome data (attrition bias), selective outcome reporting (reporting bias) and other potential sources of bias (Table 2).

### Effect of L-carnitine on plasma Lp(a) concentrations

**Figure 2:**
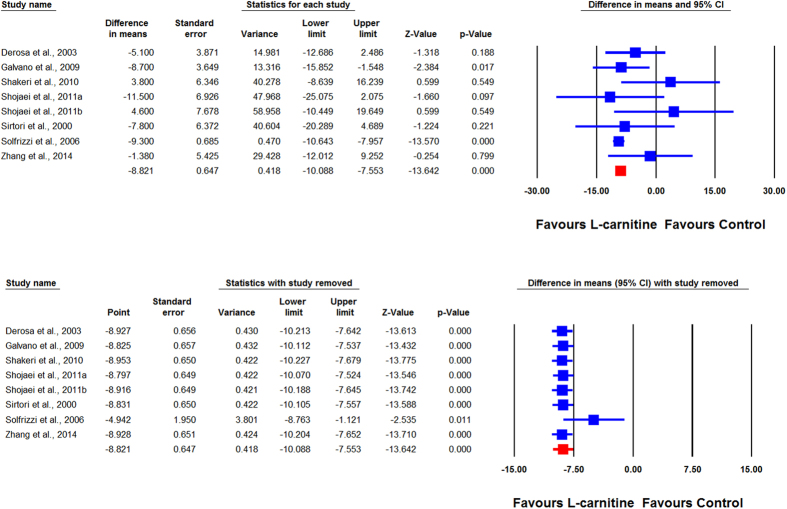
Forest plot detailing weighted mean difference and 95% confidence intervals for the impact of L-carnitine on plasma Lp(a) concentrations. Lower plot shows leave-one-out sensitivity analysis.

**Figure 3:**
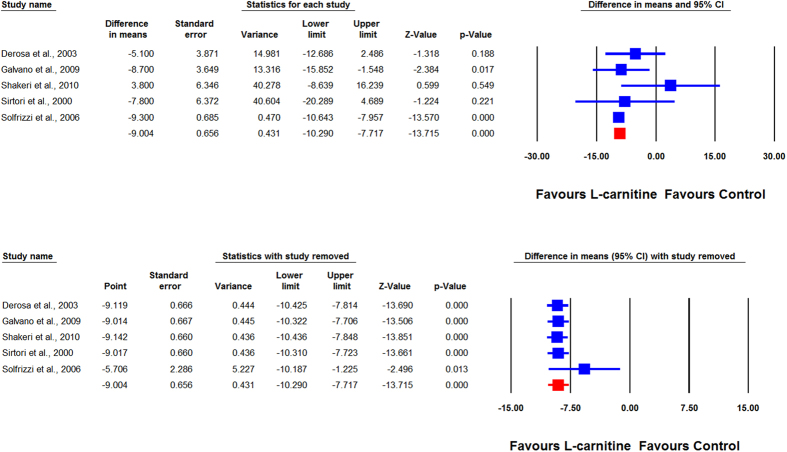
Forest plot detailing weighted mean difference and 95% confidence intervals for the impact of oral L-carnitine on plasma L(a) concentrations. Lower plot shows leave-one-out sensitivity analysis.

**Figure 4:**
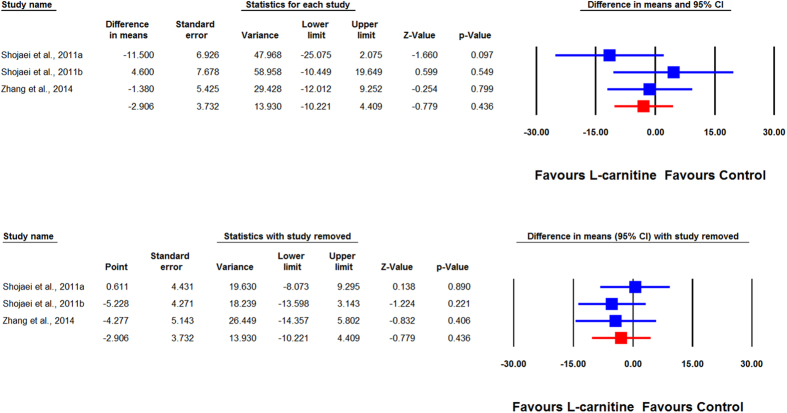
Forest plot detailing weighted mean difference and 95% confidence intervals for the impact of intravenous L-carnitine on plasma Lp (a) concentrations. Lower plot shows leave-one-out sensitivity analysis.

Overall, the impact of L-carnitine on plasma Lp(a) levels were reported in 7 trials comprising 8 treatment arms with 375 participants. Meta-analysis suggested a significant reduction of Lp(a) levels following supplementation with L-carnitine (WMD: −8.82 mg/dL, 95% CI: −10.09, −7.55, *p* < 0.001). This result was robust in the leave-one-out sensitivity analysis (Fig. 2). When the studies were categorized according to the route of administration, a significant reduction in plasma Lp(a) concentration was observed with oral (WMD: −9.00 mg/dL, 95% CI: −10.29, −7.72, *p* < 0.001) (Fig. 3) but not intravenous L-carnitine supplementation (WMD: −2.91 mg/dL, 95% CI: −10.22, 4.41, *p* = 0.436). Both of these latter effects were robust in the sensitivity analysis (Fig. 4).

### Effect of L-carnitine on plasma lipids

**Figure 5:**
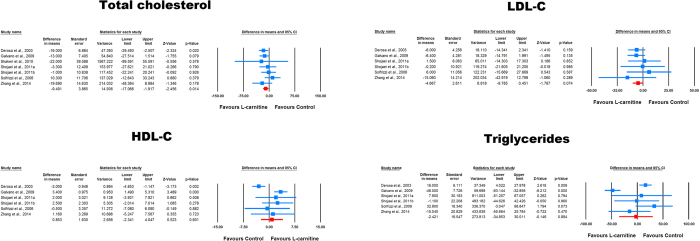
Forest plot detailing weighted mean difference and 95% confidence intervals for the impact of L-carnitine on plasma lipids.

Overall, the impact of L-carnitine on plasma total cholesterol, LDL-C, HDL-C and triglycerides levels were reported in 7, 6, 6 and 6 treatment arms, respectively. Meta-analysis suggested a significant reduction in plasma concentrations of total cholesterol (WMD: −9.49 mg/dL, 95% CI: −17.07, −1.92, *p* = 0.014) and a borderline significant trend in reduction of LDL-C (WMD: −4.67 mg/dL, 95% CI: −9.78, 0.45, *p* = 0.074) following L-carnitine supplementation (Fig. 5). However, plasma HDL-C (WMD: 0.85 mg/dL, 95% CI: −2.34, 4.05, *p* = 0.601) and triglycerides (WMD: −2.42 mg/dL, 95% CI: −34.85, 30.01, *p* = 0.884) were not altered by L-carnitine (Fig. 5).

### Meta-regression analysis

**Figure 6:**
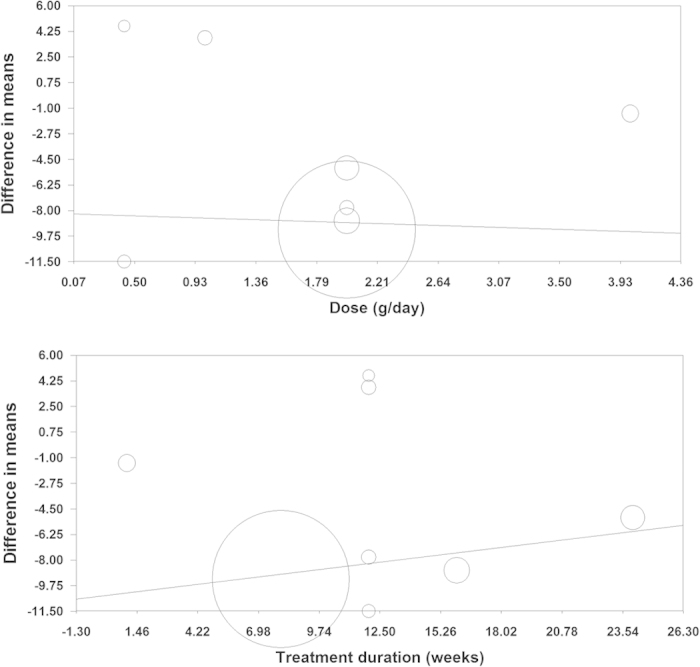
Meta-regression plots of the association between mean changes in plasma Lp(a) concentrations after L-carnitine treatment with dose and duration of treatment.

Potential associations between the Lp(a)-lowering effects of L-carnitine with dose and duration of supplementation were evaluated using meta-regression analysis. The results suggested that the pooled estimate is independent of L-carnitine dose (slope: −0.30; 95% CI: −4.19, 3.59; *p* = 0.878) and duration of treatment (slope: 0.18; 95% CI: −0.22, 0.59; *p* = 0.374) (Fig. 6).

### Publication bias

**Figure 7:**
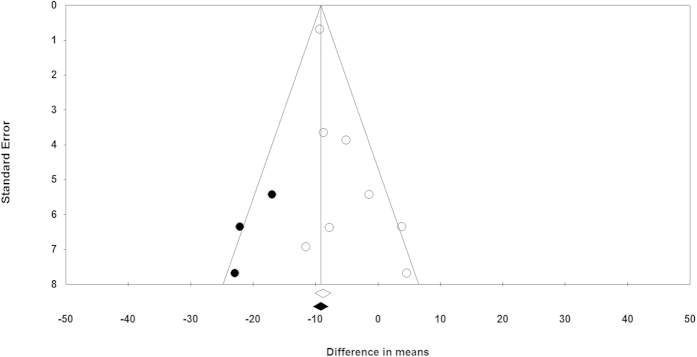
Funnel plot detailing publication bias in the studies reporting the impact of L-carnitine on plasma Lp(a) concentrations. Open diamond represents observed effect size; closed diamond represents imputed effect size.

The funnel plot of the study standard error by effect size (WMD) was asymmetric, suggesting potential publication bias that was also confirmed by the results of Egger’s linear regression test (intercept = 1.08, standard error = 0.41; 95% CI = 0.09, 2.09, *t* = 2.61, df = 6, two-tailed *p* = 0.04) (Fig. 7). However, there was no sign of bias according to the Begg’s rank correlation test (Kendall’s Tau with continuity correction = 0.32, *Z* = 1.11, two-tailed *p*-value = 0.266). An attempt was made to adjust the effect size by imputing potentially missing studies using “trim and fill” correction. This approach led to the imputation of 3 missing trials on the left side of funnel plot, yielding a corrected effect size of −9.16 mg/dL (95% CI: −10.41, −7.92) (Fig. 7). The “fail-safe N” test showed that 89 studies would be needed to bring the effect size down to a non-significant (*p* > 0.05) value.

## Discussion

To our knowledge, the current systematic review and meta-analysis is the first to analyze evidence from RCTs on the efficacy of supplementation with L-carnitine on plasma Lp(a) concentrations. The results, albeit with small numbers of subjects overall, suggest a significant reduction of Lp(a) levels following oral supplementation with L-carnitine.

L-carnitine, a derivate of the amino acid lysine, is found predominantly in red meat, certain fish, and trace levels observed in dairy and some plant foods, such as avocado^43^. The effects of L-carnitine on lipid metabolism have been investigated in many experimental and clinical studies, but the exact mechanisms responsible for L-carnitine lowering effects on Lp(a) levels are not completely understood. However, the reason for these effects could be that L-carnitine, by stimulating fatty acid break-down in mitochondria, might reduce liver production of Lp(a)^43^. An experimental study has shown that L-carnitine inhibits advanced glycation end products (AGE)-modification of bovine serum albumin more potent than aminoguanidine, a prototype inhibitor of AGE^43^. In hemodialysis patients, L-carnitine supplementation might play a protective role against vascular injury based on reduction in circulating levels of AGEs^44^.

Recent studies have showed that oral ingestion of L-carnitine in both animals and humans results in generation of trimethylamine by intestinal microbiota, which is absorbed and then metabolized to trimethylamine-N-oxide (TMAO) by liver flavin monoxygenases (FMOs) (especially FMO3 and FMO1)^35^,^45^. Since L-carnitine is contained in red meat, omnivorous humans have higher values of TMAO than vegetarians, explaining why L-carnitine and the microbiota may contribute to the causal link between high consumption of red meat and CV risk^35^. Recent metabolomics and animal studies showed that TMAO, an intestinal microbiota-dependent metabolite formed from dietary trimethylamine-containing nutrients such as phosphatidylcholine (PC), choline, and carnitine, is linked to coronary artery disease pathogenesis, only in the presence of TMA/TMAO generation and intact gut microbiota^35^,^46^. The mechanisms by which TMAO raises CV risk and accelerates atherosclerosis are not well understood, but studies have shown that TMAO arising from both choline and L-carnitine supplementation in mice may inhibit reverse cholesterol transport^35^ and enhance foam cell formation in both arterial and peritoneal cavity^47^. Direct provision of TMAO into diet has been shown to increase the development of atherosclerosis in animal models, confirming its pro-atherogenic properties^47^, and multiple clinical studies have confirmed that an elevated TMAO level is associated with increased risks for incident major adverse CV events^48^.

The levels of TMAO are modulated by hepatic FMO3, which is regulated by a bile acid-activated nuclear receptor named the farnesoid X receptor (FXR)^49^. The FMOs are known to oxidize sulfur-, phosphorus-, and selenium-containing compounds and endogenous amines, including TMA^50^. Antisense oligonucleotide mediated suppression of FMO3 levels have recently been shown to suppress TMAO levels, and to inhibit atherosclerosis in animal models^51^. Furthermore, FMO3 has been shown to serve as a central regulator of tissue cholesterol balance, and to be involved in insulin resistance and dyslipidemic changes in mouse models, suggesting the FMO3 and TMAO pathway as potential mediators of diabetes associated atherosclerosis^52^,^53^.

In contrast to the potential dangerous effects in the acceleration of atherosclerosis, mixed results have been reported with L-carnitine supplementation in the immediate setting following an acute myocardial infarction (AMI). While the major RCTs examining L-carnitine supplementation in the immediate post-MI setting showed no clinical benefit, a meta-analysis of L-carnitine intervention studies suggested its potential beneficial effect^54^. Other studies have suggested beneficial effects of L-carnitine supplementation on inflammatory parameters^55^, in the secondary prevention of CVD^54^,^56^, in diabetes mellitus^57^, on serum lipid profile in hemodialysis patients^58^, for adults with end-stage kidney disease on hemodialysis^59^ or for patients in maintenance hemodialysis^60^, though detailed analysis of whether the carnitine was provided in oral *vs* intravenous supplementation, bypassing the gut microbiota, has not been extensively evaluated. In our meta-analysis, L-carnitine significantly reduced total cholesterol and showed borderline significant reduction of LDL-C, while plasma HDL-C and triglycerides were not affected. According to the available data L-carnitine supplementation might also significantly reduce triglycerides levels, but especially when low HDL-C levels are present^61^. However, the real effects of L-carnitine supplementation, taking into account its above-mentioned positive and possibly negative properties (*via* TMAO), on the process of atherosclerosis and atheroma plaque merit further investigations.

This meta-analysis has several important limitations, and the obtained results should be viewed as suggestive only. Most notable, there were only few eligible RCTs included in this meta-analysis, and most of them had a relatively modest number of participants. Moreover, none of the RCTs included have hard clinical outcome endpoints, and the duration of the interventions was relatively modest. Furthermore, the included studies were heterogeneous concerning the characteristics of patients and study design (2 of studies on L-carnitine supplementation have been performed in the setting of hemodialysis when carnitine metabolism is altered in uremia and the depletion of carnitine is expected^62^, 4 in diabetic/metabolic syndrome patients with dyslipidemia, and 1 in hyperlipidemic patients). There was also the diversity of the co-administered medications (statins or coenzyme Q10). Moreover the duration of the L-carnitine supplementation differs from one study to another, therefore further studies are required to test the time dependence of the observed effect as well as the impact of L-carnitine bioavailability on the Lp(a)-lowering activity. Many characteristics that vary within studies, such as the type of study, the year of publication, the inclusion and exclusion criteria, the sample size, the control group or quality of the studies, could have been the factors of between-study heterogeneity. However, we used “leave-one-out” sensitivity analysis under a conservative random-effects model to assess the possibility of some bias such as unclear double-randomization or large unbalanced dropout. Our results suggest that the significance of estimated pooled effect size is not biased by any single study.

The meta-analysis suggests that L-carnitine supplementation might be associated with significant Lp(a) lowering. However, given the TMAO elevating effect of oral L-carnitine supplementation, whether or not L-carnitine is a reasonable therapy to reduce Lp(a) levels requires further long-term investigations with hard clinical endpoints such as AMI, stroke and mortality risks. Prospective trials are required to fully elucidate the clinical value of L-carnitine supplementation.

## Additional Information

**How to cite this article**: Serban, M.-C. *et al.* Impact of L-carnitine on plasma lipoprotein(a) concentrations: A systematic review and meta-analysis of randomized controlled trials.. *Sci. Rep.* **6**, 19188; doi: 10.1038/srep19188 (2016).

## Figures and Tables

**Table 1:** Demographic characteristics of the included studies.

Study		Derosa *et al.*^63^	Galvano *et al.*^64^	Shakeri*et al.*^65^	Shojaei *et al.*^66^	Sirtori *et al.*^67^	Solfrizzi *et al.*^68^	Zhang *et al.*^69^
Year		2003	2009	2010	2011	2000	2006	2014
Location		Italy	Italy	Iran	Iran	Italy	Italy	China
Design		Randomized, double-masked, placebo-controlled clinical trial,	Double-blind, randomized clinical trial	Unblinded, randomized clinical trial	Randomized double-blind placebo-controlled clinical trial	Randomized double-blind placebo-controlled clinical trial	Open, randomized, parallel-group study	Randomized, single-blind placebo controlled clinical study
Duration of study		24 weeks	16 weeks	12 weeks	12 weeks	12 weeks	60 days	1 week
Inclusion criteria		Patients with newly diagnosed (within 6 months) type 2 DM managed through dietary restriction alone and with hypercholesterolemia Lp(a) level,>30 mg/dL	Patients aged 30 – 70 years with type 2 DM, that was managed through dietary restriction alone, and with dyslipidemia (defined as LDL-C > 100 mg/dl, HDL-C < 40 mg/dl and/or tot-C > 200 mg/dl) and Lp(a) levels >20 mg/dl	Hemodialysis patients in the age range of 24 to 80 years	Maintenance hemodialysis patients with moderately high serum lipoprotein(a) levels being on treatment with atorvastatin and/or lovastatin (both 20 mg/d)	Patients with plasma Lp(a) levels ranging between 40 and 80 mg/dl, characterized by additional hyperlipidemia	Patients with type 2 DM, a triglyceride serum levels <400 mg/dL (<4.5 mmol/L), and Lp(a) serum levels >20 mg/dL (>0.71 mmol/L) at two consecutive measurements at 4 and 5 weeks from the dietary lead-in period	Overweight or obese (body mass index [BMI] 28.2 +/− 1.8 kg/m^2^) patients aged from 27–65 years with metabolic syndrome
Route of administration		oral	NR	oral	intravenous	oral	oral	intravenous
L-Carnitine Dose		2 g/day	2 g/day	1 g/day	1 g 3 times per week	2 g/day	2 g/day	4 g/day
Participants	Case	46	38^d^	18	12^a^	18	26^d^	15
					13^b^			
					14^c^			
	Control	48	37^e^	18	13	18	26^d^	15
Age (years)	Case	52 (6)	52.1 ± 8.1^d^	54.5 ± 19.0	55.3 ± 15.6^a^	55.1 ± 7.4	65.4 ± 10.4^d^	46.9 ± 9.14
					53.5 ± 11.5^b^			
					52.8 ± 10.4^c^			
	Control	50 (7)	51.4 ± 7.6^e^	57.0 ± 20.0	51.6 ± 19.2	57.8 ± 11.2	62.7 ± 8.74^e^	46.8 ± 10.9
Male (%)	Case	52.2	39.47^d^	67	50^a^	66.6	50^d^	26.6
					53.8^b^			
					42.8^c^			
	Control	47.9	40.54^e^	61	46.1	50	53.8^e^	33.3
BMI (kg/m^2^)	Case	27.3 (2.5)	27.8 ± 2.0^d^	23 ± 4	24.3 ± 2.1^a^	NR	29 (24.5–33)^d^	28.2 ± 1.8
					23.6 ± 2.4^b^			
					23.3 ± 2.3^c^			
	Control	26.8 (2.2)	27.1 ± 2.4^e^	23 ± 3	23.6 ± 2.6	NR	27.8 (23.5–31)^e^	27.1 ± 2.3
hs-CRP (mg/L)	Case	NR	NR^d^	NR	NR^a^	NR	NR^d^	3.06 ± 1.69
					NR^b^			
					NR^c^			
	Control	NR	NR^e^	NR	NR	NR	NR^e^	2.3 ± 3.74
Total cholesterol (mg/dL)	Case	235 (30)	261 ± 21^d^	NR	147.1 ± 28.8^a^	277.1 ± 33.5	225.6 ± 35.4^d^	208.04 ± 35.57
					150.9 ± 25.6^b^			
					152.5 ± 23.5^c^			
	Control	228 (4 I)	259 ± 24^e^	NR	156.5 ± 31.9	262.3 ± 28.1	239.9 ± 57.9^e^	206.49 ± 44.08
LDL-C (mg/dL)	Case	161 (16)	162.8 ± 16.4^d^	NR	84.5 ± 26.5^a^	NR	144.3 ± 34.6^d^	146.17 ± 37.12
					89.5 ± 28.7^b^			
					83.2 ± 32.2^c^			
	Control	157 (19)	161.4 ± 18.1^e^	NR	84.0 ± 13.9	NR	157 ± 54.2^e^	143.85 ± 41.76
HDL-C (mg/dL)	Case	45 (4)	38.4 ± 0.8^d^	NR	41.9 ± 7.8^a^	57 ± 17.4	41.2 ± 10.7^d^	42.92 ± 10.05
					40.9 ± 3.2^b^			
					39.4 ± 7.0^c^			
	Control	43 (5)	38.2 ± 0.7^e^	NR	42.8 ± 5.7	50.9 ± 13.4	41.1 ± 12.2^e^	44.47 ± 8.50
Triglycerides (mg/dL)	Case	125 (35)	301 ± 24^d^	NR	149.2 ± 75.2^a^	138.8 ± 38.3	189.5 ± 55.4^d^	152.34 ± 68.20
					131.0 ± 53.2^b^			
					155.9 ± 62.6^c^			
	Control	156 (28)	298 ± 32^e^	NR	150.9 ± 76.9	139.5 ± 54.5	209.7 ± 61.7^e^	149.69 ± 61.11
Glucose (mg/dL)	Case	135 (30)	136 ± 27^d^	NR	NR^a^	NR	NR^d^	93.69 ± 10.99
					NR^b^			
					NR^c^			
	Control	141 (25)	137 ± 28^e^	NR	NR	NR	NR^e^	91.89 ± 16.57
SBP (mmHg)	Case	NR	149 ± 12^d^	NR	NR^a^	NR	NR^d^	120.6 ± 15.45
					NR^b^			
					NR^c^			
	Control	NR	150 ± 11^e^	NR	NR	NR	NR^e^	135.7 ± 15.6
DBP (mmHg)	Case	NR	80 ± 11^d^	NR	NR^a^	NR	NR^d^	78.9 ± 9.4
					NR^b^			
					NR^c^			
	Control	NR	79 ± 12^e^	NR	NR	NR	NR^e^	80.8 ± 8.4
Lp(a) (mg/dL)	Case	29.6 (I 8.3)	29.7 ± 11.2^d^	76 ± 37	45.0 ± 23.9	45.4 ± 17.8	41 (34.0–64.0)^d^	23.34 ± 9.04
					48.6 ± 22.8			
					50.3 ± 18.4			
	Control	27.8 (20.2)	29.5 ± 10.8^e^	61 ± 26	31.6 ± 7.7	54.1 ± 17.4	36.5 (25.3–49.0)^e^	27.34 ± 9.14

Values are expressed as mean ± SD or median (25–75 percentiles). ABBREVIATIONS: BMI: body mass index; Nr: not reported; LDL-C: low-density lipoprotein cholesterol; HDL-C: high-density lipoprotein cholesterol; SBP: systolic blood pressure; DBP: diastolic blood pressure; hs-CRP: high-sensitivity C-reactive protein; BMI: body mass index; DM: diabetes mellitus; ^a^denotes 1000 mg (L-carnitine, Carnivore, Sigma tau, Spain), 3 times per week, after each dialysis session; ^b^denotes oral coenzyme Q10, 100 mg/d (Pharmed-Tnt Inc, Canada); ^c^denotes a combinations of both carnitine and coenzyme Q10; ^d^denotes L-carnitine 2 g/day and simvastatin 20 mg; ^e^denotes 20 mg/day simvastatin tablet.

**Table 2:** Assessment of risk of bias in the included studies using Cochrane criteria.

Study	Ref	Sequence gene-ration	Allocation concea-lment	Blinding of partici-pants and person-nel	Blinding of outcome assessment	Incom-plete outcome data	Selec-tive out-come repor-ting	Other poten-tial threats to validity
Derosa *et al.*	63	L	U	L	L	L	L	L
Galvano *et al.*	64	H	U	L	L	L	L	L
Shakeri *et al.*	65	U	U	H	H	L	L	L
Shojaei *et al.*	66	L	U	L	L	L	L	L
Sirtori *et al.*	67	L	U	L	L	L	L	L
Solfrizzi *et al.*	68	U	U	H	H	L	L	L
Zhang *et al.*	69	L	H	L	L	L	L	L

L: low risk of bias; H: high risk of bias; U: unclear risk of bias.
